# Increased risk of active tuberculosis during pregnancy and postpartum: a register-based cohort study in Sweden

**DOI:** 10.1183/13993003.01886-2019

**Published:** 2020-03-19

**Authors:** Jerker Jonsson, Sharon Kühlmann-Berenzon, Ingela Berggren, Judith Bruchfeld

**Affiliations:** 1 Dept of Public Health Analysis and Data Management, The Public Health Agency of Sweden, Solna, Sweden; 2 Division of Infectious Diseases, Dept of Medicine Solna, Karolinska Institutet, Stockholm, Sweden; 3 Dept of Communicable Disease Control and Prevention, Stockholm County Council, Stockholm, Sweden; 4 Dept of Infectious Diseases, Karolinska University Hospital, Stockholm, Sweden

## Abstract

**Rationale:**

Studies investigating the risk of active tuberculosis (TB) in association with pregnancy have not been conclusive. We aimed to investigate this risk in a large retrospective register-based cohort study in Sweden.

**Methods:**

Data from women of 15–49 years of age who had given birth in Sweden between 2005 and 2013 were extracted from the national childbirth register and linked to the national TB register. Cohort time was divided into three exposure periods: during pregnancy, six months (180 days) postpartum and time neither pregnant nor postpartum. We calculated incidence rates (IRs) per 100 000 person-years for each period and incidence rate ratios (IRRs) with IRs neither pregnant nor postpartum as the reference.

**Results:**

The cohort included 649 342 women, of whom 553 were registered as cases of active TB, 389 when neither pregnant nor postpartum, 85 during pregnancy and 79 when postpartum. Overall IRs were 9, 12 and 17 cases per 100 000 person-years, respectively, giving IRR 1.4, 95% CI 1.1–1.7 (during pregnancy) and IRR 1.9, 95% CI 1.5–2.5 (when postpartum). Stratification by TB incidence in country of origin showed that the increased risk was concentrated amongst women from countries with a TB incidence of 100 or higher, where IRs per 100 000 person-years were 137 (when neither pregnant nor postpartum), 182 (during pregnancy) and 233 (when postpartum).

**Conclusion:**

We show a significant increase in risk of active TB during both pregnancy and postpartum in women from high incidence countries and recommend TB screening in pregnant women belonging to this risk group.

## Abstract 2

**There is an increased risk of active TB in women during pregnancy and postpartum. We recommend screening for latent TB in pregnant women from TB risk groups and for clinicians to use a low threshold for suspecting active TB in pregnancy and postpartum** http://bit.ly/34pqXT8

## Introduction

Until the middle of the 20th century, active tuberculosis (TB) during pregnancy was considered a serious complication of both conditions [1] and, as a consequence, sometimes an indication for termination of pregnancy. In addition, women who had survived TB were often dissuaded from having children. With the introduction of effective TB medication this changed [2, 3] and, after adequate treatment of TB in women of reproductive age, no increased risk of relapse during pregnancy has been observed [4].

During pregnancy there is, among other cytokine changes, a significant decrease in tumour necrosis factor-α (TNF-α) secretion from NK-cells. These changes occur gradually over the course of the pregnancy and tend to be more pronounced in the 2nd and 3rd trimesters [5]. There is an increased risk of TB activation associated with treatment by TNF-α inhibitors [6, 7], illustrating the importance of TNF-α in containing *Mycobacterium tuberculosis* infection.

Studies investigating an increased risk of active TB in association with pregnancy have not been conclusive [8–10]. While some studies have shown no increased risk or even a reduced risk of active TB during pregnancy, a study from 2012 showed an increased risk, particularly during postpartum (defined as within 6 months after delivery) [9]. The increased risk of active TB postpartum has been interpreted as possibly due to delayed diagnosis, as symptoms of active TB can be mistaken for symptoms related to the pregnancy itself. In addition, symptoms might not appear until after delivery due to unmasking of TB when a normal immune response is restored [11–14]. A delayed diagnosis of active TB is a danger not only to the pregnant woman but also to the newborn child [15–17]. Transmission *in utero* is considered to be rare [18] and the main risk to the child from a mother with pulmonary TB occurs after birth. An early diagnosis of active TB before delivery is therefore important to reduce this risk. Screening for latent tuberculosis infection (LTBI) during pregnancy could also be a good opportunity for prevention if pregnancy and postpartum constitute an increased risk of activating LTBI.

Sweden is a low TB incidence country (5.3 per 100 000 inhabitants in 2017) [19] and the majority of TB cases are diagnosed in individuals originating from high TB incidence countries, likely infected before migrating to Sweden. The risk of active TB decreases with time in Sweden due to the reduced risk of new exposure when living in a low incidence country.

Few large studies on risk of TB in association with pregnancy have been conducted [9] and prospective cohort studies are both costly and time-consuming. In Sweden, national registers with excellent coverage over time are available for pregnancies and childbirth [20], as well as for active TB [21], immigration and death [22], and can be linked through unique personal identity numbers. The aim of this retrospective study was to investigate, using available registers, if pregnancy and postpartum constitute risk factors for active TB.

## Methods

This was a national retrospective register-based cohort study. The cohort consisted of all women of reproductive age, defined as between 15 and 49 years old, who gave birth at least once in Sweden during the study period (January 01, 2005 to December 31, 2013). There was no programmatic TB screening in pregnant women in Sweden during the study period, regardless of risk group. *A priori* we calculated, in a one-sided two-sample Poisson test, that a yearly TB incidence among pregnant women of at least 7.0 per 100 000 was sufficient to detect a significant difference in the population yearly TB incidence in Sweden of 6.0 per 100 000 inhabitants with 80% power, 5% significance level and given an average of 100 000 births per year.

Women contributed time from the date they turned 15 years old or the date of immigration to Sweden if they were older than 15 years. Time contribution ended when the first of the following occurred: date they turned 50 years old, date they emigrated from Sweden, death, or end of study period. We excluded women with an incomplete identity number and those lacking date of birth or date of delivery.

The cohort was grouped according to TB incidence in the respective country of birth, based on the average of World Health Organization (WHO) country estimates during the study period [23] (supplementary table S1) as follows: 1) low incidence: less than 25 cases per 100 000 inhabitants per year; 2) medium incidence: 25 to 99 cases per 100 000 inhabitants per year; and 3) high incidence: 100 or more cases per 100 000 inhabitants per year.

The time contributed by each woman was divided into three periods of exposure: ‘pregnancy’, ‘postpartum’ (180 days after delivery) and ‘time neither pregnant nor postpartum’. If a new pregnancy started before the end of the 180 days postpartum of the previous birth, the subsequent time was counted as during pregnancy.

**FIGURE 1:**
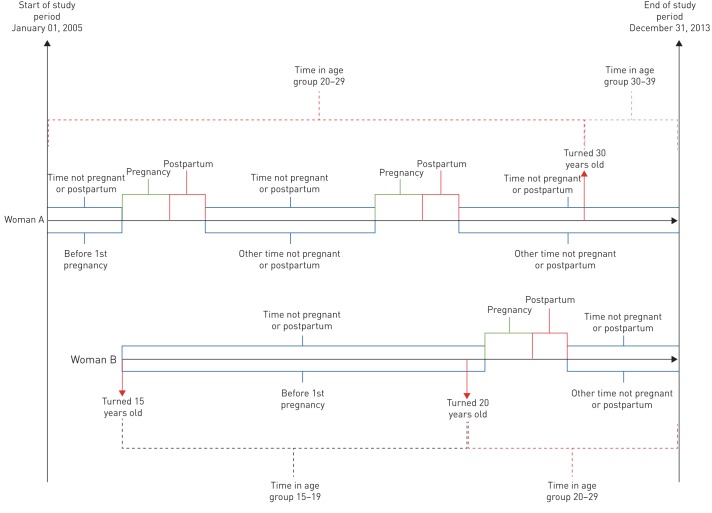
Timeline for the study of tuberculosis (TB) risk in pregnant women aged 15–49 years in Sweden for the period 2005–2013, with two examples of how contribution of time was stratified. Time was divided into three different periods: ‘pregnancy’, ‘postpartum’ and ‘time not pregnant or postpartum’. In the analysis of women from high incidence countries, ‘time not pregnant or postpartum’ was divided further into two periods: ‘before 1st pregnancy’ and ‘other time not pregnant or postpartum’, and also stratified by age group. Woman A was 22 years old at the start of the study and contributed time to age group 20–29 until she turned 30 years old, whereupon she contributed to age group 30–39. Woman B entered the study when she turned 15 years old and contributed time to age group 15–19 until she turned 20, whereupon she contributed to age group 20–29.

For the women from high incidence countries, we also investigated the influence of time in Sweden for the risk of active TB, assuming that the majority of them had been exposed to TB before immigration to Sweden and that their risk of active TB was highest during their first years in the country whether pregnant or not. In order to test this hypothesis, the time neither pregnant nor postpartum was divided into two parts, time ‘before 1st pregnancy’ (*i.e.* time from when first included in the study period) and ‘other time not pregnant or postpartum’ (figure 1). In this group of women the time contributed was also stratified by the age groups 15–19, 20–29, 30–39 and 40–49 years.

### Registers

The cohort was extracted from the Swedish medical birth register [20] held at the National Board of Health and Welfare, which includes all registered births in Sweden occurring later than gestational week 22. The register contains information on date of birth and country of birth of the mother, as well as date of delivery and estimated length of pregnancy.

Data on TB diagnosis was extracted from the TB register of SmiNet [21] at the Public Health Agency of Sweden (PHAS), which contains all reported cases of active TB. Laboratory confirmed cases have a positive culture of *M. tuberculosis* or a sample with positive microscopy for acid-fast bacteria paired with a positive PCR test for the *M. tuberculosis* complex. For cases with no laboratory confirmation, the clinical criteria for reporting are symptoms and/or radiological findings consistent with active TB and a clinician's decision to treat with a full course of TB medication. For cases with no date of diagnosis reported, the date of the first laboratory report was used as a proxy. For cases lacking laboratory confirmation, we used the date of the clinical report if date of diagnosis was lacking.

The mother's country of origin, dates of immigration, dates of emigration and date of death were obtained from the Swedish Population Register at Statistics Sweden [22]. For asylum seekers, the date of immigration registered did not always correspond to the date of entering Sweden as registration had been delayed for some women. This was evident in 14 women registered as giving birth in Sweden before the official date of immigration, which was then changed to 1 day before giving birth. The same occurred in nine cases of active TB diagnosed in Sweden before their official date of immigration, which was then set to the immigration date stated in the TB register.

All data were linked using the Swedish unique national identity number. In women lacking a Swedish national identity number, a temporary identity number given for health care matters was used. After linkage, Statistics Sweden deleted the identity numbers before data was analysed at PHAS.

### Statistical methods

The incidence rates (IRs) were calculated as the number of TB events per person-time-at-risk for each of the exposure periods. The incidence rate ratios (IRRs) were calculated as the IR during pregnancy or postpartum divided by the IR during time not pregnant or postpartum. Both IRs and IRRs were reported with 95% confidence intervals (CIs) and considered statistically significant if the CI did not include one. In the analysis of the subgroup of women from high incidence countries, the IRRs were calculated as the IR during time before 1st pregnancy, pregnancy and postpartum divided by the IR during other time not pregnant or postpartum.

Statistical analysis was conducted using R statistical software version 3.4.1 (www.r-project.org) [24].

### Complementary analysis of background population

In order to confirm that the results were reasonable, we compared them with an estimate of IR for TB in the different exposure periods for the population of all women aged 15–49 years in Sweden (also including all women who did not give birth during the study period). Population data per year was retrieved from Statistics Sweden to obtain person-years and the total number of TB cases among women in this age group was obtained from the TB register at PHAS.

### Ethical considerations

The regional ethics committee in Stockholm granted ethical permission (Dnr 2014/1504–31/3) and waived the need for informed consent as the study was retrospective and only used anonymised data from already existing registers.

## Results

### Main analysis

The total number of registered deliveries in Sweden between January 01, 2005 and December 31, 2013 was 951 672. After applying the exclusion criteria the final cohort consisted of 649 342 women contributing time during the study period, with a total of 951 530 deliveries (table 1). The average time contribution of each woman was 8.5 person-years, of which 1.1 person-years were during pregnancy and 0.7 person-years were postpartum.

**TABLE 1:** Number of women and total time contributed (in person-years), as stratified by tuberculosis (TB) incidence in the country of origin.

**TB incidence in country of origin**	**Subjects**	**Time contributed** **person-years**
**Low (less than 25 cases per 100** **000 population)**	546 980	4 792 201
**Medium (25–99 cases per 100** **000 population)**	57 326	426 995
**High (100 cases or more per 100** **000 population)**	44 536	305 249
**Unknown origin**	500	3677
**Total**	649 342	5 528 112

The mean age at first delivery during the study period was 29.9 years (median 30 years, range 15–49 years) and the mean number of deliveries per woman was 1.5 (range 1–7).

During the study period, 553 of the women were reported as diagnosed with active TB, of which 452 (82%) were verified by mycobacterial culture. There were 85 cases diagnosed during pregnancy, 79 during postpartum and 389 when not pregnant or postpartum (table 2).

**TABLE 2:** Number of tuberculosis (TB) cases in women during ‘pregnancy’, ‘postpartum’ or ‘time not pregnant or postpartum’, with incidence rates (IRs) and incidence rate ratios (IRRs), as stratified by TB incidence in the country of origin.

**TB incidence in country of origin**	**Exposure period**	**TB cases (n=553)**	**Time contributed** **person-years**	**IR (95% CI) per 100** **000** **person-years**	**IRR (95% CI)**
**All women**	Pregnancy	85	689 288	12 (10–15)	**1.4** **(1.1–1.7)**
	Postpartum	79	456 102	17 (14–21)	**1.9** **(1.5–2.5)**
	Time not pregnant or postpartum	389	4 382 732	8.9 (8.1–9.9)	1.0 (reference)
**Low (less than 25 cases per 100** **000 population)**	Pregnancy	1	584 836	0.17 (0.04–0.63)	0.20 (0.03–1.49)
	Postpartum	2	386 420	0.52 (0.16–1.44)	0.62 (0.15–2.58)
	Time not pregnant or postpartum	32	3 820 945	0.84 (0.59–1.15)	1.00 (reference)
**Medium (25–99 cases per 100** **000 population)**	Pregnancy	2	58 862	3.4 (1.1–9.5)	0.25 (0.06–1.05)
	Postpartum	7	39 343	18 (9–33)	1.3 (0.6–3.0)
	Time not pregnant or postpartum	44	328 790	13 (10–18)	1.00 (reference)
**High (100 cases or more per 100** **000 population)**	Pregnancy	82	45 081	182 (147–223)	**1.32** **(1.04–1.69)**
	Postpartum	70	30 017	233 (184–290)	**1.70** **(1.31–2.20)**
	Time not pregnant or postpartum	313	230 151	137 (123–153)	1.00 (reference)
**Unknown origin**	Pregnancy	0	509	0	–
	Postpartum	0	322	0	–
	Time not pregnant or postpartum	0	2846	0	–

Values in bold are statistically significant.

**FIGURE 2:**
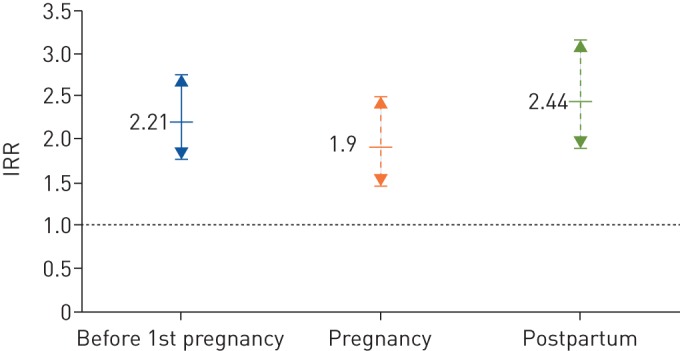
Tuberculosis (TB) incidence rate ratios (IRRs) with 95% CIs in women from high TB incidence countries. Categories: ‘before 1st pregnancy’, ‘pregnancy’ and ‘postpartum’ (as compared to ‘other time not pregnant or postpartum’).

There was an overall significantly increased risk of active TB both during pregnancy (IRR 1.4, 95% CI 1.1–1.7) and postpartum (IRR 1.9, 95% CI 1.5–2.5) compared to when not pregnant or postpartum (table 2). When stratifying by incidence in country of birth, the increased risk was only confirmed in women from high incidence countries and corresponded to TB IRs per 100 000 person-years of 182 (pregnancy), 233 (postpartum) and 137 (not pregnant or postpartum) (table 2). Of the 313 cases diagnosed when not pregnant or postpartum, 172 (55%) were diagnosed before the start of the 1st pregnancy (figure 2).

**FIGURE 3:**
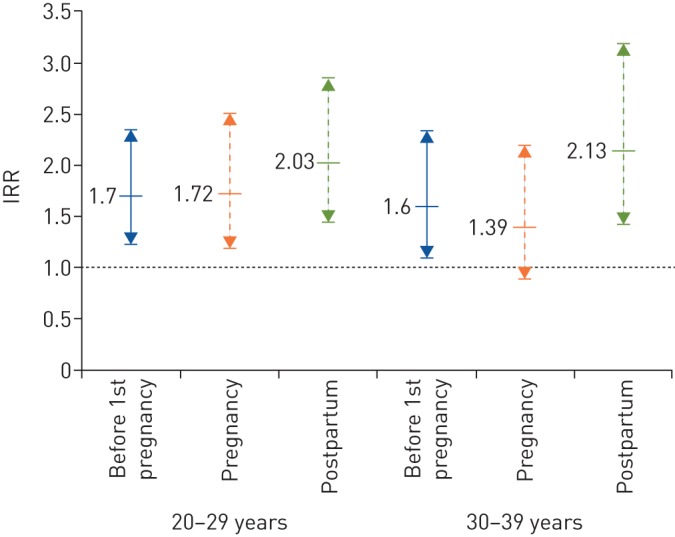
Tuberculosis (TB) incidence rate ratios (IRRs) with 95% CIs in women from high TB incidence countries, as stratified by age group (20–29 and 30–39 years). Categories: ‘before 1st pregnancy’, ‘pregnancy’ and ‘postpartum’ (as compared to ‘other time not pregnant or postpartum’).

When stratifying the time contributed by women from high incidence countries by age group, IRRs for all exposures were statistically significant for age groups 20–29 years and 30–39 years, with the exception of the age group 30–39 years during pregnancy, which was borderline. Although all confidence intervals overlapped with each other, there was a tendency towards a higher IRR postpartum as compared to before 1st pregnancy (supplementary table S2 and figure 3).

### Complementary analysis of background population

The complementary analysis of the background population showed a significant increase in risk of active TB during pregnancy and an even higher increase in risk during postpartum compared to during time not pregnant or postpartum (table 3). The person-years included in the study cohort constituted 29% of the person-years from the background population.

**TABLE 3:** Number of tuberculosis (TB) cases in all women in Sweden aged 15–49 years, with incidence rates (IRs) and incidence rate ratios (IRRs), as stratified by the exposure period

**Exposure period**	**TB cases**	**Time contributed** **person-years**	**IR (95% CI) per 100** **000** **person-years**	**IRR (95% CI)**
**Pregnancy**	85	689 213	12.3 (9.9–15.3)	**1.6 (1.5–1.7)**
**Postpartum**	79	456 364	17.3 (13.7–21.6)	**2.3 (2.2–2.4)**
**Time not pregnant or postpartum**	1339	17 738 509	7.6 (7.2–8.0)	1.0 (reference)
**Total**	1503	18 884 086	8.0 (7.6–8.4)	–

Values in bold are statistically significant.

## Discussion

In this study we show a significantly increased risk of active TB during pregnancy and when postpartum in a cohort of women aged 15–49 years who had given birth in Sweden, a low incidence setting (6.8 per 100 000 population average during the study period). Even though the overall TB IRs were low, we validated these findings by analysis of the total Swedish population of women aged 15–49 years during the same study period, which resulted in similar IRRs but with more narrow confidence intervals.

When stratifying by incidence in country of birth, these risks were significant in women originating from countries with a high TB incidence (*i.e.* more than 100 cases per 100 000 inhabitants per year). In women from low TB incidence countries (less than 25 cases per 100 000), of which the vast majority were born in Sweden, the scarcity of cases resulted in incidences of less than one per 100 000 for all periods.

Our results confirm findings from a study in the United Kingdom by Zenner *et al.* [9], which demonstrated an increased risk of active TB in women postpartum, although an increased risk during pregnancy could not be shown. A stronger statistical power in our study is a possible explanation for this difference, with 553 TB cases included in our study overall as compared to 177 TB cases in the study by Zenner *et al*. A more recent European cross-sectional study also concluded that TB was diagnosed more frequently after delivery [25] and possible reasons stated were delayed diagnosis due to pregnancy or late or poor attendance at antenatal care that might be more common in risk groups for TB.

During the study period, almost 90% of diagnosed TB cases in Sweden were born outside of the country and the majority of them originated from high incidence countries [19]. In our study, 465 out of 553 of the TB cases (84%) were born in high incidence countries. According to observations from national TB surveillance [19], the majority of active TB cases in migrants are diagnosed within the first 5 years of arrival in Sweden. We could confirm this observation among women from high incidence countries aged 20–29 and 30–39 years old, where we showed a significantly increased IRR for active TB before 1st pregnancy, but also during pregnancy and postpartum, as compared to other time not pregnant or postpartum. In a low incidence setting like Sweden, the risk for re-infection after completed treatment, for LTBI or active TB, is very low and, thus, the women diagnosed with TB before pregnancy had a much reduced risk of developing active TB again, pregnant or not.

Maternal health controls represent an excellent opportunity to screen women belonging to risk groups for TB, in order to initiate early diagnosis and treatment of active TB or initiate preventive therapy when indicated, as has been recommended elsewhere [26, 27]. Mother and child will benefit from an early diagnosis of both active TB and LTBI, preferably well before the child is born. As LTBI is a continuous source of new cases of active TB, LTBI treatment is an important part of the WHOs End TB Strategy [28], which aims for a 90% reduction in the TB IR by 2035 (as compared to 2015). Consequently, the WHO issued guidelines on LTBI screening and treatment in 2014, with a recent update in 2017. However, TB screening during pregnancy or postpartum is not recommended in these guidelines [29]. Our data provide robust evidence that women from high TB prevalence countries are at substantially elevated risk of developing active TB in both pregnancy and during the postpartum period. We believe that introduction of routine LTBI screening in this group, preferably in early pregnancy, should be considered by public health bodies in countries with low TB prevalence. Notably, it is far more probable that active TB in pregnancy is the result of LTBI activation/progression than of re-activation of adequately treated active TB, although definitive proof is currently lacking.

### Limitations

The national childbirth register we used does not include pregnancies shorter than 22 weeks and may reflect a reduced time period during pregnancy. However, it is unlikely that this biased the results since immunological changes during pregnancy, something that was not investigated here and which theoretically could explain the increased risk of TB activation, are more pronounced during the last two trimesters [5].

The register data did not include any information on other health conditions that might have influenced the risk of developing active TB, such as HIV, being underweight, or nutritional deficiencies. Neither was there any information on possible factors that might have increased the risk of TB exposure, like, for example, time spent in refugee camps or imprisonment. However, in Sweden all pregnant women are offered screening for HIV [30] and, to our knowledge, there has been no report of active TB and HIV co-morbidity in pregnancy to date.

We had no means to control for events like earlier pregnancies or TB diagnosis occurring outside of Sweden before immigration. As the risk of exposure is greatly reduced after moving to a low TB incidence country, we believe our results underestimate the added risk of active TB during pregnancy and postpartum.

Almost all pregnant women in Sweden are in contact with the national health program of antenatal and postnatal care. This introduces a possible risk of bias in TB diagnosis while pregnant and postpartum due to closer contact with health services as compared to when not pregnant or postpartum. In most cases though, the contact with health services focuses more on the woman during pregnancy compared to postpartum. However, TB incidence was higher postpartum, which contradicts a possible bias.

Information on preventive treatment for LTBI in our study cohort is lacking. As the largest target group for LTBI treatment in Sweden is recent immigrants from high incidence countries, the effect of LTBI treatment in our cohort could possibly lead to a lower TB incidence per 100 000 person-years in this group; however, this should not affect the IRRs.

For those women who had events registered in Sweden (TB diagnosis or childbirth) before their official immigration date, we corrected the erroneous dates of immigration from Statistics Sweden. For the remainder, less time not pregnant or postpartum might have been registered, possibly increasing the reference incidence and thus underestimating the increase in risk of active TB associated to pregnancy.

### Conclusions

Our findings of an increased risk of active TB during pregnancy and postpartum in women from high TB incidence countries is probably generalisable to other settings. In addition, an even more pronounced increase is likely in a high incidence setting where there is a continuous risk of renewed TB exposure. Active TB during pregnancy and when postpartum poses severe risks for both the mother and the child. Our study adds knowledge to a controversial field and we consequently recommend screening for LTBI in pregnant women from TB risk groups, such as women from high TB incidence countries or with known recent TB exposure. For any woman, being of child-bearing age increases the motivation for chemoprophylaxis in latently infected individuals. Clinicians should apply a low threshold when considering active TB and investigate accordingly in pregnant women, as well as in women in the postpartum period, who belong to TB risk groups.

## Supplementary material

10.1183/13993003.01886-2019.Supp1 **Please note:** supplementary material is not edited by the Editorial Office, and is uploaded as it has been supplied by the author.
Supplementary table S1 ERJ-01886-2019.table_S1
Supplementary table S2 ERJ-01886-2019.table_S2

## Shareable PDF

10.1183/13993003.01886-2019.Shareable1 This one-page PDF can be shared freely online.
Shareable PDF ERJ-01886-2019.Shareable
