# Supplementary material for: Increased risk of active tuberculosis during pregnancy and postpartum: a register-based cohort study in Sweden
Source: Eur Respir J. 2020 Mar 19;55(3):1901886. doi: 10.1183/13993003.01886-2019 (PMC7083553; doi:10.1183/13993003.01886-2019)
Supplement: Supplementary file 1 [file ERJ-01886-2019.table_S1.pdf]

Country	average TB incidence per year, 2005 to 2013	Group
Albania	17	1. Low (< 25)
Antigua and Barbuda	6,2	1. Low (< 25)
Australia	6,4	1. Low (< 25)
Austria	10	1. Low (< 25)
Bahamas	14	1. Low (< 25)
Barbados	2,4	1. Low (< 25)
Belgium	10	1. Low (< 25)
Canada	5,2	1. Low (< 25)
Chile	17	1. Low (< 25)
Costa Rica	14	1. Low (< 25)
Cuba	7,9	1. Low (< 25)
Cyprus	5,0	1. Low (< 25)
Czechia	7,9	1. Low (< 25)
Denmark	7,1	1. Low (< 25)
Dominica	13	1. Low (< 25)
Egypt	19	1. Low (< 25)
Fiji	24	1. Low (< 25)
Finland	6,7	1. Low (< 25)
France	9,2	1. Low (< 25)
Germany	6,4	1. Low (< 25)
Greece	5,7	1. Low (< 25)
Grenada	2,8	1. Low (< 25)
Hungary	16	1. Low (< 25)
Iceland	4,1	1. Low (< 25)
Iran	17	1. Low (< 25)
Ireland	10	1. Low (< 25)
Israel	5,9	1. Low (< 25)
Italy	7,1	1. Low (< 25)
Jamaica	4,9	1. Low (< 25)
Japan	21	1. Low (< 25)
Jordan	7,1	1. Low (< 25)
Lebanon	13	1. Low (< 25)
Luxembourg	7,7	1. Low (< 25)
Malta	10	1. Low (< 25)
Mauritius	22	1. Low (< 25)
Mexico	21	1. Low (< 25)
Monaco	2,0	1. Low (< 25)
Netherlands	6,9	1. Low (< 25)
New Zealand	8,0	1. Low (< 25)
Norway	7,3	1. Low (< 25)
Oman	13	1. Low (< 25)
Poland	23	1. Low (< 25)
Saint Lucia	9,3	1. Low (< 25)
Samoa	12	1. Low (< 25)
Saudi Arabia	16	1. Low (< 25)
Seychelles	19	1. Low (< 25)
Slovakia	11	1. Low (< 25)
Spain	17	1. Low (< 25)

Sweden	6,8	1. Low (< 25)
Switzerland	7,1	1. Low (< 25)
Syrian Arab Republic	24	1. Low (< 25)
Tonga	15	1. Low (< 25)
Trinidad and Tobago	20	1. Low (< 25)
United Arab Emirates	2,5	1. Low (< 25)
United Kingdom of Great Britain and Northern Ireland	15	1. Low (< 25)
United States of America	4,4	1. Low (< 25)
Uruguay	24	1. Low (< 25)
West Bank and Gaza Strip	1,08	1. Low (< 25)
Algeria	75	2. Medium (25-99)
Argentina	26	2. Medium (25-99)
Armenia	66	2. Medium (25-99)
Bahrain	27	2. Medium (25-99)
Belarus	69	2. Medium (25-99)
Benin	68	2. Medium (25-99)
Brazil	45	2. Medium (25-99)
Bulgaria	41	2. Medium (25-99)
Burkina Faso	59	2. Medium (25-99)
China	82	2. Medium (25-99)
China	82	2. Medium (25-99)
Colombia	31	2. Medium (25-99)
Dominican Republic	70	2. Medium (25-99)
Ecuador	62	2. Medium (25-99)
El Salvador	34	2. Medium (25-99)
Estonia	31	2. Medium (25-99)
Guatemala	29	2. Medium (25-99)
Honduras	54	2. Medium (25-99)
Iraq	46	2. Medium (25-99)
Jugoslavia countries; Slovenien, Serbien, Bosnien, Makedonien, Kroatien and Montenegro	29	2. Medium (25-99)
Kuwait	30	2. Medium (25-99)
Latvia	57	2. Medium (25-99)
Libya	40	2. Medium (25-99)
Lithuania	75	2. Medium (25-99)
Malaysia	76	2. Medium (25-99)
Maldives	42	2. Medium (25-99)
Mali	64	2. Medium (25-99)
Nicaragua	52	2. Medium (25-99)
Palau	69	2. Medium (25-99)
Panama	56	2. Medium (25-99)
Paraguay	43	2. Medium (25-99)

Portugal	30	2. Medium (25-99)
Qatar	40	2. Medium (25-99)
Republic of Korea	94	2. Medium (25-99)
Rwanda	89	2. Medium (25-99)
Singapore	38	2. Medium (25-99)
Solomon Islands	91	2. Medium (25-99)
Sri Lanka	66	2. Medium (25-99)
Togo	73	2. Medium (25-99)
Tunisia	30	2. Medium (25-99)
Turkey	27	2. Medium (25-99)
Venezuela	29	2. Medium (25-99)
Yemen	59	2. Medium (25-99)
Afghanistan	189	3. High (>100)
Angola	379	3. High (>100)
Azerbaijan	179	3. High (>100)
Bangladesh	225	3. High (>100)
Bhutan	192	3. High (>100)
Bolivia	140	3. High (>100)
Botswana	563	3. High (>100)
Burundi	156	3. High (>100)
Cabo Verde	148	3. High (>100)
Cambodia	452	3. High (>100)
Cameroon	272	3. High (>100)
Central African Republic	491	3. High (>100)
Chad	151	3. High (>100)
Congo	399	3. High (>100)
Cote d'Ivoire	212	3. High (>100)
Democratic People's Republic of Korea	375	3. High (>100)
Democratic Republic of the Congo	327	3. High (>100)
Djibouti	529	3. High (>100)
Equatorial Guinea	126	3. High (>100)
Eritrea	112	3. High (>100)
Ethiopia	282	3. High (>100)
Gabon	503	3. High (>100)
Gambia	181	3. High (>100)
Georgia	138	3. High (>100)
Ghana	186	3. High (>100)
Guinea	192	3. High (>100)
Guyana	115	3. High (>100)
Haiti	239	3. High (>100)
India	254	3. High (>100)
Indonesia	420	3. High (>100)
Kazakhstan	153	3. High (>100)
Kenya	313	3. High (>100)
Kyrgyzstan	163	3. High (>100)
Lao People's Democratic Republic	232	3. High (>100)

Liberia	288	3. High (>100)
Madagascar	246	3. High (>100)
Malawi	344	3. High (>100)
Mauritania	145	3. High (>100)
Mongolia	428	3. High (>100)
Morocco	100	3. High (>100)
Mozambique	539	3. High (>100)
Myanmar	388	3. High (>100)
Namibia	715	3. High (>100)
Nepal	163	3. High (>100)
Niger	120	3. High (>100)
Nigeria	340	3. High (>100)
Pakistan	276	3. High (>100)
Papua New Guinea	432	3. High (>100)
Peru	137	3. High (>100)
Philippines	325	3. High (>100)
Republic of Moldova	168	3. High (>100)
Romania	116	3. High (>100)
Russian Federation	110	3. High (>100)
Senegal	137	3. High (>100)
Sierra Leone	316	3. High (>100)
Somalia	286	3. High (>100)
South Africa	938	3. High (>100)
Sudan	109	3. High (>100)
Swaziland	1220	3. High (>100)
Tajikistan	144	3. High (>100)
Thailand	192	3. High (>100)
Timor-Leste	498	3. High (>100)
Turkmenistan	120	3. High (>100)
Uganda	215	3. High (>100)
Ukraine	113	3. High (>100)
United Republic of Tanzania	441	3. High (>100)
Uzbekistan	102	3. High (>100)
Viet Nam	160	3. High (>100)
Zambia	516	3. High (>100)
Zimbabwe	452	3. High (>100)
