# Supplementary material for: Increased risk of active tuberculosis during pregnancy and postpartum: a register-based cohort study in Sweden
Source: Eur Respir J. 2020 Mar 19;55(3):1901886. doi: 10.1183/13993003.01886-2019 (PMC7083553; doi:10.1183/13993003.01886-2019)
Supplement: Supplementary file 2 [file ERJ-01886-2019.table_S2.pdf]

Table S.2. Number of TB cases, person-years, incidence rates and incidence rate ratios with confidence intervals; before 1st pregnancy, during pregnancy and postpartum compared to other time not pregnant or postpartum, in women from high TB incidence countries, by age groups, Sweden 2005-2013.

Age group	Risk period	No of TB diagnosis (n=468)	Person-years	Incidence rate per 100 000 person-years (95% CI)	Incidence rate ratio (95% CI)
**All age groups**	Before pregnancy	175	82 687	212 (181-254)	**2.21** (1.77-2.76)
	During pregnancy	82	45 081	182 (145-226)	**1.90** (1.45-2.50)
	Postpartum	70	30 018	233 (182-295)	**2.44** (1.90-3.14)
	Other time not pregnant or postpartum	141	147 511	96 (80-113)	1.00 reference
**15-19 years**	Before pregnancy	21	6 885	305 (189-466)	0.55 (0.16-1.83)
	During pregnancy	2	975	205 (25-741)	0.37 (0.06-2.19)
	Postpartum	2	461	434 (53-1567)	0.78 (0.18-3.26)
	Other time not pregnant or postpartum	3	536	560 (115-1636)	1.00 reference
**20-29 years**	Before pregnancy	108	43 606	248 (203-299)	**1.70** (1.23-2.34)
	During pregnancy	53	21 128	251 (188-328)	**1.72** (1.18-2.50)
	Postpartum	39	13 134	297 (211-406)	**2.03** (1.45-2.85)
	Other time not pregnant or postpartum	56	38 354	146 (110-190)	1.00 reference
**30-39 years**	Before pregnancy	43	31 460	137 (99-184)	**1.60** (1.10-2.33)
	During pregnancy	25	21 092	119 (77-175)	1.39 (0.88-2.19)
	Postpartum	27	14 858	182 (120-264)	**2.13** (1.42-3.19)
	Other time not pregnant or postpartum	72	84 281	85 (67-108)	1.00 reference
**40-49 years**	Before pregnancy	3	736	408 (84-1191)	**9.92** (2.73-36.1)
	During pregnancy	2	1 887	106 (13-383)	2.58 (0.57-11.8)
	Postpartum	2	1 565	128 (16-462)	3.11 (0.72-13.5)
	Other time not pregnant or postpartum	10	24 340	41 (20-76)	1.00 reference
