# Supplementary material for: Increased risk of active tuberculosis during pregnancy and postpartum: a register-based cohort study in Sweden
Source: Eur Respir J. 2020 Mar 19;55(3):1901886. doi: 10.1183/13993003.01886-2019 (PMC7083553; doi:10.1183/13993003.01886-2019)
Supplement: Supplementary file 3 [file ERJ-01886-2019.Shareable.pdf]

ORIGINAL ARTICLE
TUBERCULOSIS

SHAREABLE PDF

Increased risk of active tuberculosis during pregnancy and postpartum: a register-based cohort study in Sweden

Jerker Jonsson [1,2], Sharon Kühlmann-Berenzon [1], Ingela Berggren [2,3] and Judith Bruchfeld [2,4]

Affiliations: [1]Dept of Public Health Analysis and Data Management, The Public Health Agency of Sweden, Solna, Sweden. [2]Division of Infectious Diseases, Dept of Medicine Solna, Karolinska Institutet, Stockholm, Sweden. [3]Dept of Communicable Disease Control and Prevention, Stockholm County Council, Stockholm, Sweden. [4]Dept of Infectious Diseases, Karolinska University Hospital, Stockholm, Sweden.

Correspondence: Jerker Jonsson, Dept of Public Health Analysis and Data Management, The Public Health Agency of Sweden, SE-171 82 Solna, Sweden. E-mail: jerker.jonsson@folkhalsomyndigheten.se

@ERSpublications
There is an increased risk of active TB in women during pregnancy and postpartum. We recommend screening for latent TB in pregnant women from TB risk groups and for clinicians to use a low threshold for suspecting active TB in pregnancy and postpartum http://bit.ly/34pqXT8

Cite this article as: Jonsson J, Kühlmann-Berenzon S, Berggren I, *et al.* Increased risk of active tuberculosis during pregnancy and postpartum: a register-based cohort study in Sweden. *Eur Respir J* 2020; 55: 1901886 [https://doi.org/10.1183/13993003.01886-2019].

This single-page version can be shared freely online.

ABSTRACT

Rationale: Studies investigating the risk of active tuberculosis (TB) in association with pregnancy have not been conclusive. We aimed to investigate this risk in a large retrospective register-based cohort study in Sweden.

Methods: Data from women of 15–49 years of age who had given birth in Sweden between 2005 and 2013 were extracted from the national childbirth register and linked to the national TB register. Cohort time was divided into three exposure periods: during pregnancy, six months (180 days) postpartum and time neither pregnant nor postpartum. We calculated incidence rates (IRs) per 100 000 person-years for each period and incidence rate ratios (IRRs) with IRs neither pregnant nor postpartum as the reference.

Results: The cohort included 649 342 women, of whom 553 were registered as cases of active TB, 389 when neither pregnant nor postpartum, 85 during pregnancy and 79 when postpartum. Overall IRs were 9, 12 and 17 cases per 100 000 person-years, respectively, giving IRR 1.4, 95% CI 1.1–1.7 (during pregnancy) and IRR 1.9, 95% CI 1.5–2.5 (when postpartum). Stratification by TB incidence in country of origin showed that the increased risk was concentrated amongst women from countries with a TB incidence of 100 or higher, where IRs per 100 000 person-years were 137 (when neither pregnant nor postpartum), 182 (during pregnancy) and 233 (when postpartum).

Conclusion: We show a significant increase in risk of active TB during both pregnancy and postpartum in women from high incidence countries and recommend TB screening in pregnant women belonging to this risk group.

Copyright ©ERS 2020. This article is open access and distributed under the terms of the Creative Commons Attribution Licence 4.0.
